# Surveillance of acute flaccid paralysis in the Marches region (Italy): 1997–2007

**DOI:** 10.1186/1471-2334-8-135

**Published:** 2008-10-09

**Authors:** Marcello M D'Errico, Pamela Barbadoro, Sonia Bacelli, Elisabetta Esposto, Vania Moroni, Federica Scaccia, Luana Tantucci, Emilia Prospero, the AFP Study Group

**Affiliations:** 1 Institute of Infectious Diseases and Public Health, Università Politecnica delle Marche, Italy; 2 Chair of Hygiene, Institute of Infectious Diseases and Public Health, Università Politecnica delle Marche Piazza Roma 2, 60100 – Ancona, Italy

## Abstract

**Background:**

The last case of poliomyelitis due to transmission of indigenous wild poliovirus occurred in Italy in 1982, however, it is important to guarantee a high quality surveillance as there is a risk of importation of cases from areas where polio is endemic. Stopping poliovirus transmission is pursued through a combination of high infant immunization coverage and surveillance for wild poliovirus through reporting and laboratory testing of all cases of acute flaccid paralysis (AFP) among children under fifteen years of age. The aim of this study was to describe and to evaluate 11 years of active surveillance in the Marches (Italy) in terms of: incidence, aetiology and clinical manifestation of AFP cases.

**Methods:**

The active Acute Flaccid Paralysis surveillance has been carried out in the Marches region since February 1997 by the Chair of Hygiene which established a regional hospital network. Active surveillance involves 15 hospital centres.

**Results:**

In the considered period, 0–15 years population varied between 187,051 in 1997 to 201,625 in 2007, so the number of AFP expected cases is 2 per year. From February 1997 to October 2007, 27 cases were found with rates of 1.0/100,000 in 1997; 2.0/100,000 in 1998; 1.0/100,000 in 1999; 0.5/100,000 in 2000; 2.5/100,000 in 2001; 1.0/100,000 in 2002; 0 in 2003; 0.5/100,000 in 2004; 1.5/100,000 in 2005; 2.0/100,000 in 2006; 1.5/100,000 in 2007. In 29.6% of cases two stool samples were collected in 14 days from the symptoms onset. The 60-days follow-up is available for 23 out of 27 cases reported. In 44.5% of cases the definite diagnosis was Guillain Barrè syndrome.

**Conclusion:**

In general, the surveillance activity is satisfactory even if in presence of some criticalities in biological samples collection. The continuation of surveillance, in addition to the maintenance of current levels of performance, will tend to a further and more detailed sensitization of all workers involved, in order to obtain spontaneous and prompt reporting, and to achieve the optimal standards recommended by the WHO both in the collection of biological samples and the availability of 60 days follow-up, with the goal of eradicating polio from all countries.

## Background

In May 1988, the World Health Assembly committed the World Health Organization (WHO) to achieving the goal of global eradication of poliomyelitis by the year 2000. This goal is defined as:

• no cases of clinical poliomyelitis associated with wild poliovirus, and

• no wild poliovirus found worldwide [1].

Europe has been poliofree since 2002, the last case of poliomyelitis due to transmission of indigenous wild poliovirus occurred in Italy in 1982 [2,3]. Despite the problem of poliomyelitis is not a priority, in poliofree countries, it is important to guarantee a high quality surveillance as there is a risk of importation of cases from areas where the disease is endemic. The year 2006 began with confirmation that indigenous wild poliovirus transmission had been stopped in Egypt and Niger, reducing the number of endemic countries to a historic low of four (in 1988, wild poliovirus was endemic in more than 125 countries). Four countries had not interrupted indigenous transmission of wild poliovirus: Afghanistan, India, Nigeria and Pakistan and accounted for 94% of all new cases of poliomyelitis in 2006. The remaining 6% of all new cases of poliomyelitis occurred in countries into which poliovirus has been reintroduced [4,5].

Poliomyelitis (Polio) is a highly infectious disease caused by a virus. It invades the nervous system, and can cause total paralysis in a matter of hours. It can strike at any age, but affects mainly children under three (over 50% of all cases). One in 200 infections leads to irreversible paralysis (usually in the legs). Amongst those paralysed, 5–10% die when their breathing muscles become immobilized. Although polio paralysis is the most visible sign of polio infection, fewer than 1% of polio infections ever result in paralysis. Poliovirus can spread widely before cases of paralysis are seen. Because of this silent transmission and the rapid spread of poliovirus, WHO considers a single confirmed case of polio paralysis to be evidence of an outbreak. There is no cure for polio, it can only be prevented through immunization. Polio vaccine, given multiple times, almost always protects a child for life. Full immunization will markedly reduce an individual's risk of developing paralytic polio. Polio is one of only a limited number of diseases that can be eradicated, this is because polio only affects humans, an effective vaccine is available, and immunity is lifelong. [6,7] Stopping poliovirus transmission has been pursued through a combination of routine immunization and supplementary immunization campaigns which are guided by high quality surveillance. The four key strategies for stopping poliovirus transmission are:

1. **High infant immunization coverage **with four doses of oral polio vaccine (OPV) in the first year of life, WHO has established a global target of at least 90% immunization coverage for all vaccines used in the Expanded Programme on Immunization, including oral polio vaccine, by the year 2000. In Italy, since 2002 polio immunization schedule has been changed from two doses of OPV and two of IPV to 4 doses of the last one, OPV has been excluded.

2. **National immunization days (NIDs): **this is a supplementary immunization which is intended to complement, not replace, routine immunization. The aim of mass campaigns is to interrupt circulation of poliovirus by immunizing every child under 5 years of age with two doses of OPV, regardless of previous immunization status.

3. **Surveillance for wild poliovirus **through reporting and laboratory testing of all cases of acute flaccid paralysis (AFP) among children under fifteen years of age. The number of cases reported each year is used as an indicator of a country's ability to detect polio, even in countries where the disease no longer occurs. A country's surveillance system should be sensitive enough to detect at least 1 case of AFP for every 100,000 children under 15, even in the absence of polio and if are tested ≥ 80% of stool specimens.

4. **Targeted "mop-up" campaigns **are implemented in a country when the final pockets of poliovirus transmission have been identified with certification-standard surveillance. The campaigns involve door-to-door immunization in high-risk districts where the virus is known or suspected to still be circulating.

AFP case was defined as a child aged less than 15 years showing acute onset of flaccid paralysis in one or more limbs, or acute onset of bulbar paralysis [8,2]. The differential diagnosis of acute flaccid paralysis includes paralytic poliomyelitis, Guillain-Barré syndrome and transverse myelitis; less common aetiologies are traumatic neuritis, encephalitis, meningitis and tumours. Distinguishing characteristics of paralytic polio are: asymmetric flaccid paralysis, fever at onset, rapid progression of paralysis, residual paralysis after 60 days, and preservation of sensory nerve function [7-9].

The aim of this study was to describe and to evaluate 11 years of active surveillance in the Marches (Italy) in terms of: incidence, aetiology and clinical manifestation of AFP cases.

## Methods

### Organization of AFP Surveillance in Italy

In January 1996, a program of active AFP surveillance with a target population aged 0–15 was set-up as a pilot study limited to four representative regions of Italy located in different parts of the country. The surveillance, extended to a national level in 1997, is conducted by the National Institute of Health (ISS) and the Ministry of Health (MOH) through reference laboratories, mainly University Laboratories of Hygiene, located in each of the 20 Italian regions. Local networks of collaborating hospitals (Paediatrics, Neurology and Infectious Disease Wards) were directly coordinated by the person in charge of the Regional Reference Centre (RRC).

Every case of AFP in the 0–15 years population has to be notified to ISS and MOH by the RRC through a preliminary questionnaire with clinical and epidemiological information.

Clinical samples for virological investigation are collected for each case and consist of: two stool specimens taken 24–48 hours apart and one serum sample, within 14 days since the onset of paralysis, samples are appropriately placed in refrigerated container for transport. Laboratory analysis consists of the evaluation of viral growth after two blind passages on cells lines (RD and L20B) sensitive to poliovirus (3 serotypes) infection and non polio enterovirus (*Coxsackie virus *A; *Coxsackie virus *B1, B2, B3, B6; *Echovirus*; *Enterovirus *70, 71) infection. Negativity refers to the absence of viral growth.

All tests were carried out according to the standard protocols recommended by WHO.

Serum samples are tested only if stool specimens are potentially infectious for poliovirus. After the notification, a follow-up questionnaire with more detailed information has to be sent to ISS and MOH to determine whether there is residual paralysis or death at 60 days after diagnosis, and to clarify the final diagnosis (card concerning the case containing information about clinical, instrumental and laboratory examinations).

Cases are classified based on clinical and laboratory information, in:

1. **poliomyelitis**: poliovirus identified in the stool specimen;

2. **non polio**: cases without poliovirus in stool specimens and no residual paralysis at 60 days;

3. **polio compatible**: cases not definitely classified as non-polio on the available information. Acute disease with residual paralysis after 60 days or death or loss of the subject during follow-up, in which it was not possible to collect at least 2 stool samples within 14 days from the onset of paralytic symptoms;

4. **vaccine-associated paralytic poliomyelitis (VAPP)**: paralytic disease caused by vaccination with OPV. The association is thought to be valid if the vaccine is administered at the same subject in a period of time from 4 to 30 days before the onset of symptoms. As for contacts a period of 4–75 days must be considered. In patients with immunodeficiency the period may be longer than 30 days for recipients cases and longer than 75 days for the contact cases [10].

### Organization of AFP Surveillance in the Marches Region

The Chair of Hygiene at the Università Politecnica of the Marche, a reference Centre for Marche region, has started the surveillance of acute flaccid paralysis in February 1997 realizing a network of 15 hospital centres, placed in the 4 provinces of the Marche. One or more references have been identified in each hospital centre. Every 15–20 days all reference doctors of the 25 Operative Units (15 of Paediatrics, 3 of Infectious Diseases, 7 of Neurology/Child Neuropsychiatry) belonging to the network are contacted by phone. For every case of AFP the management is activated according to the directives of ISS and MOH.

This surveillance did not expect the authorization from a local Ethics Committee as it is a national ministerial project taking into account the WHO directives for the achievement of the poliomyelitis eradication (WHA 41.28 of May 1988). This work has been realised in part thanks to the financial contribution of the research project "Prevention of risk factors of the mother-child health". Art 12 D.L.vo. 502/1992, research line 4.4, "Study of flaccid paralysis in Italy". Stool and serum samples are collected form children suffering from acute flaccid paralysis subject to parents or a legal guardian agreement taken by a doctor of the department. This consensus is included in the medical record of every patient. Research carried out on humans was performed in compliance with the Helsinki Declaration.

## Results and discussion

In the Marche Region 0–15 years population varied from 187,051 in 1997 to 201,625 in 2007, so the number of AFP expected cases is 2 per year.

**Figure 1:**
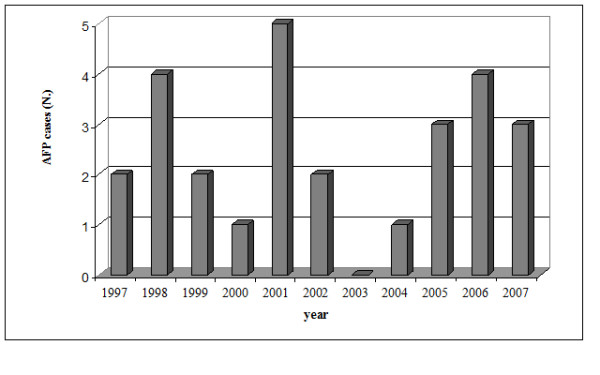
Distribution of AFP cases in the Marches region (from February 1997 to December 2007).

Between February 1997 and December 2007, 27 cases were reported (Figure 1), with rates of 1.0/100,000 in 1997; 2.0/100,000 in 1998; 1.0/100,000 in 1999; 0.5/100,000 in 2000; 2.5/100,000 in 2001; 1.0/100,000 in 2002; 0 in 2003; 0.5/100,000 in 2004; 1.5/100,000 in 2005; 2.0/100,000 in 2006; 1.5/100,000 in 2007.

The mean age of AFP cases was 7 years (SD, ± 4 years): 37% of cases were less 5 years of age, 37% between 5 and 10 years and 26% between 10 and 15 years. There is a similar proportion of AFP cases by sex: 15 in males and 12 in females.

The distribution by month is characteristic, with 70.4% of cases notified between May and October.

Considering the Province of residence of cases, these are distributed with a percentage of 59.3% in the south of the Region. 44.4% of case reports comes from the High Specialized Hospital "G Salesi" (Child Neuropsychiatry, Paediatrics and Paediatric Intensive Care Units).

The final diagnosis of AFP in the 27 reported cases is shown in table 1. No case of poliomyelitis and vaccine-associated paralytic poliomyelitis was identified. Guillain-Barrè syndrome (GBS) was the most common diagnosis (44.5%), followed by polyradiculoneuritis (7.4%), encephalomyelitis/myelitis (18.5%), 1 case immunitary multineuropathy, 1 case by synovial cyst compression. Cases with residual disabilities at 60 days were 7 out of 23. In two cases no diagnostic definition was found (but a 60–90 days follow up permitted to exclude paralysis); four cases were lost to follow up, one faecal specimen was analysed for each of these cases, with no poliovirus isolation. One of these cases was classified as non polio by the MOH on the basis of medical records."

**Table 1:** Final classification in 27 cases of AFP (from February 1997 to December 2007).

**Final classification**	**No. cases (%)**
Guillain-Barrè syndrome	12
Encephalomyelitis/myelitis	5
Polyradiculuneuritis	2
Synovial cyst compression	1
Immunitary multineuropaty	1
Lost during follow up	4
No diagnostic definition	2

All patients showed serious motor deficiency due to paralytic involvement of the limbs; in 7 cases at the beginning of the paralysis was present fever, 17 cases showed the progression of paralysis within 4 days from the onset of symptoms, in 19 cases paralysis was symmetric and in 4 cases occurred a respiratory paralysis.

At follow-up, that was performed after 60–90 days from the onset of paralytic symptoms, 4 cases were lost. In 16/23 cases was found a normal resumption of walking about, while in 7/23 were reported some disabilities as for example limb and goose like walk. Three cases of AFP were also reported in outpatients: two from other Italian regions and one from Germany. The only case reported in the year 2000 was related to a case of AFP reported by a Primary Care Paediatrician. One case of 2007 was reported from a Paediatric Hospital of another Italian Region.

As for laboratory tests, in general stool samples were collected in 77.8% cases. Only one stool sample was collected in 5/21 cases and two stool samples were collected from 16/21 cases.

All faecal specimens analyzed by the reference laboratory (National Institute of Health, Rome) resulted negative for poliovirus. As reported in Table 2, the surveillance index, that is the product between case incidence and percentage of cases with a stool sample within 14 days from the symptoms onset, has had a variable course, and meeting the WHO target (= 80%) in 2002 only. In Table 2 are showed some quality indicators of the surveillance and the results reported.

**Table 2:** Quality surveillance indicators of the AFP in the Marches (from February 1997 to December 2007).

**INDICATORS**	1997	1998	1999	2000	2001	2002	2003	2004	2005	2006	2007
Incidence rate (reported cases/expected cases)	1 2/2	2 4/2	1 2/2	0.5 1/2	2.5 5/2	1 2/2	- 0/2	0.5 1/2	1.5 3/2	2 4/2	1.5 3/2

% AFP cases with follow-up at 60–90 days from report	100	75	50	100	100	100	-	100	100	75*	67

% AFP cases with initial clinical classification	100	100	100	0	100	100	-	100	100	100	100

% AFP cases with final clinical classification	100	75	0	0	100	50	-	100	100	75	67

% AFP cases with 1 stool sample within 14 days	0	50	50	0	20	100	-	0	33	25	67

% AFP cases with 2 stool samples within 14 days	0	50	50	0	0	100	-	0	33	25	33

% AFP with 2 stool samples at any time	0	75	100	0	60	100	-	0	67	75	33

% AFP with 1 stool sample at any time	50	25	0	0	20	0	-	0	0	0	67

% AFP 0 stool sample	50	0	0	100	20	0	-	100	33	25	0

**SURVEILLANCE INDEX **incidence X (% AFP cases with a sample within 14 days)	0	1	0.5	0	0.5	1	-	0	0.5	0.5	1

* missing follow-up is referred to one case archived as non-polio by the MOH after clinical charts review.

In our experience, the AFP incidence rate in the 0–15 years old population, was in general quite satisfactory.

The result obtained means that a quite good active surveillance was performed, even if with minimal yearly fluctuations in the number of cases identified. As for geographical distribution of the reporting sites, was found a predictable higher concentration of reports from the Highly Specialized Hospital "G. Salesi", as it is a reference hospital for children's diseases. The other reports, coming from hinterland structures of the Marche, show also a satisfying sensitization of all workers involved, that on several occasions actively reported the cases, anticipating the phone contact and reporting also cases managed in outpatients setting by Primary Care Paediatricians.

As for gender of reported cases (substantial equivalence between males and females) and distribution for age class (frequency slightly higher in age class 0–5 y.o.) it was not possible to make any epidemiological consideration due to the few data available. Temporal distribution of cases is rather characteristic, as most cases were concentrated in the period May-October, this situation could be related to the epidemiological progress of enterovirus infections, that in temperate climate countries spread mainly during summer and autumn. [11]

In most cases the following clinical conditions as Guillain-Barrè, Syndrome, encephalitis/meningoencephalitis and polyradiculoneuritis and other conditions are the cause of the reported flaccid paralysis [2,12]. These findings are in line with the diagnostic classification of overall reported cases internationally and nationally.

With reference to surveillance quality indicators (Table 2) some criticalities emerge compared to the values recommended by the WHO and the ISS.

The target recommended by the WHO is to obtain a percentage of AFP cases with two stool samples, collected within 14 days from the onset of symptoms, equal or higher than 80%. In Marche Region this target was achieved only in 2002; but considering the collection of two samples at any time the performance is better.

The percentage of cases with follow-up at 60–90 days is good as it is equal to 100 in 6 out of 11 years; this implies a good percentage of final clinical diagnosis.

Although emphasizing the importance of active surveillance of AFP cases, to the light of the above criticisms complementary forms of surveillance must be taken into consideration to achieve a more stable and rigorous polio eradication. First of all immunization status and vaccination coverage of indigenous and emigrants must be surveilled, moreover considering that Italy is a borderline country. In fact, a gap in immunization status [13] or the presence of parts of population not immunized [14] together with the circulation of wild poliovirus, may led to outbreaks. Italy is exposed to the risk of introduction of polio virus, because of the arrival of people from countries in which polio is still endemic. To overcome this risk, our Region has been involved from January 2008, in the National project "Surveillance of immunization status for polio of people 0–14 years" with the aim of evaluating the impact of the introduction of the IPV only vaccination schedule in order to assess vaccination coverage and proportion of people with antibodies titer against polio > 1/8. Polio outbreak can be caused by circulating vaccine derived poliovirus, as those reported in Egypt [15], Hispaniola [16] and Philippines [17] so, also if the OPV has been discontinued in Italy, people from foreign countries might contribute to the spread of these viruses, in these cases environmental surveillance of polio virus might be the key to its control.

Isolation of wild-type polioviruses from sewage samples has been shown to reflect its circulation in the community both qualitatively and quantitatively and has been used for evaluation of the effectiveness of immunization or for epidemiological investigations, in addition, such surveillance can be used to monitor the circulation of vaccine- derived polioviruses. [18] The possible implications of poliovirus capsid recombination events for the generation of potentially dangerous vaccine-derived strains are not clear at present. [19]

Italian National Institute of Health is currently participating in the WHO study with the aim of typing and characterizing of polioviruses and other enteroviruses isolated from healthy children, immunodeficient subjects and environmental samples" in six Italian cities (Palermo, Bari, Roma, Sassari, Parma e Milano). [20]

## Conclusion

In conclusion, AFP surveillance remains the gold standard of poliovirus surveillance and all efforts should be made to maintain it at high level of performance and improve it when necessary [11]. The continuation of surveillance, in addition to the maintenance of current levels of performance, will tend to a further and more detailed sensitization of all workers involved, in order to obtain spontaneous and prompt reporting, and to achieve the optimal standards recommended by the WHO also in the collection of biological samples, with the goal of eradicating polio from all countries. Other strategies to eradicate polio are needed, our Region is currently participating in the Italian MOH project for surveillance of immunization status i the 0–14 years population, environmental surveillance is not currently performed in our Region.

## Abbreviations

AFP: Acute Flaccid paralysis; GBS: Guillain-Barrè syndrome; ISS: Istituto Superiore di Sanità; MOH: Ministry of Health; NIDs: National immunization days; OPV: oral polio vaccine; RRC: Regional Reference Centre; VAPP: vaccine associated paralytic poliomyelitis; WHO: World Health Organization

## Competing interests

The authors declare that they have no competing interests.

## Authors' contributions

MMDE has made substantial contributions to conception and design of the study and in general supervision of the research group. PB has contributed in analysis and interpretation of data and has been involved in revising the manuscript. SB, LT, VM, FS, EE have contributed to acquisition, analysis and interpretation of data and in the writing of manuscript draft. EP have been involved in revising the manuscript critically for important intellectual content and in general supervision of the research group. All authors read and approved the final manuscript.

## Pre-publication history

The pre-publication history for this paper can be accessed here:


